# Maternal Immune Activation Leads to Lasting Sex-Specific Changes in Behavior and Hippocampal Morphological and Molecular Features in 12-Month-Old Rat Offspring

**DOI:** 10.3390/ijms27188222

**Published:** 2026-09-15

**Authors:** Alexandra V. Sentyabreva, Alexander S. Lyamtsev, Ekaterina A. Melnikova, Gleb M. Aliper, Anna M. Kosyreva

**Affiliations:** 1 Avtsyn Research Institute of Human Morphology of “Petrovsky National Research Centre of Surgery”, 117418 Moscow, Russia; 2 Research Institute of Molecular and Cellular Medicine, Peoples’ Friendship University of Russia (RUDN University), 117198 Moscow, Russia

**Keywords:** maternal immunity activation, sex differences, microglia, hippocampus, behavior, long-term effect

## Abstract

Maternal immune activation (MIA) during pregnancy is a recognized risk factor for neurodevelopmental and neurodegenerative disorders, including Alzheimer’s disease. Although the long-term consequences of prenatal inflammation have been extensively investigated, the influence of biological sex on age-related behavioral and hippocampal alterations remains insufficiently understood. MIA was induced in pregnant Wistar rats by the intraperitoneal administration of lipopolysaccharide (100 μg/kg) on gestational day 17. Male and female offspring were examined at 12 months of age. Behavioral patterns were assessed using the Morris water maze and elevated plus maze. Hippocampal neuronal morphology and microglial and astrocytic phenotypes were evaluated by histology and IHC, while the expression of genes associated with inflammation, neurodegeneration, neuroplasticity, oxidative stress, and neurogenesis was determined by RT-qPCR. Prenatal LPS exposure produced persistent behavioral alterations in both sexes but with distinct phenotypes. Both male and female offspring exhibited increased anxiety-like behavior, whereas females showed reduced exploratory activity during spatial memory testing. Female offspring demonstrated more pronounced hippocampal pathology, including an increased relative number of pyknotic hyperchromic neurons, a reduced ramified-to-amoeboid microglia ratio, decreased *Bdnf*, and selective upregulation of *Bace1* and *Usp11*, potentially indicating enhanced susceptibility to neurodegenerative processes. In contrast, males maintained predominantly ramified microglia morphology and showed increased expression of the neuronal progenitor gene *Pax6*, possibly suggesting the activation of compensatory mechanisms. Both sexes exhibited hippocampal upregulation of *Il18*, *Arg1*, *Mmp9*, *Ntrk2*, and *Gpx4*, consistent with chronic pro-inflammatory background accompanied by adaptive antioxidant and anti-inflammatory responses. Prenatal immune activation induces long-lasting behavioral, morphological, and molecular alterations that persist into middle age and exhibit marked sexual dimorphism. Female offspring appear more vulnerable to pro-neurodegenerative changes, whereas male offspring activate compensatory neuroprotective pathways that might partially preserve hippocampal function despite sustained pro-inflammatory reactions. These findings highlight sex as a critical biological variable in the long-term consequences of prenatal inflammation and may facilitate the identification of early biomarkers and therapeutic targets for MIA-associated neuropsychiatric and neurodegenerative disorders.

## 1. Introduction

Maternal immune activation (MIA) resulting from infectious diseases during pregnancy is considered one of the major mechanisms increasing the risk of neuropsychiatric and neurodegenerative disorders in offspring [1]. The administration of lipopolysaccharide (LPS), a component of the outer membrane of Gram-negative bacteria, to pregnant rodents is widely used to model MIA because it induces systemic inflammation resembling infection-associated inflammatory responses in humans during critical periods of central nervous system (CNS) development [2]. Experimental studies have demonstrated that prenatal immune activation induces persistent inflammatory processes in the offspring’s CNS, which may persist throughout life, impair cognitive function, and promote the development of neurodegenerative disorders [3,4,5].

The hippocampus, part of the brain’s limbic system responsible for cognitive functions, plays a pivotal role in the consolidation and retrieval of episodic and spatial memory, including linking individual elements of experience into coherent memories. It is involved in spatial navigation, learning, contextual information processing, and cognitive flexibility, enabling behavioral adaptation to changing environmental conditions [6]. Owing to the high level of activity of the processes occurring within it, the hippocampus is particularly vulnerable to adverse influences, including MIA [7]. Experimental studies of MIA demonstrate that prenatal exposure to pathogen-associated molecular patterns (PAMPs) induces both structural and functional alterations in the hippocampus of offspring. These changes include neuronal loss, impaired synaptic plasticity, disrupted neurogenesis, and the increased expression of pro-inflammatory cytokines, with the severity of these alterations progressively increasing with age. Similar findings have been reported in Sprague–Dawley rats examined at 3, 10, and 20 months of age [3], as well as in mouse models ranging from 1 to 22 months of age [4,5,8,9,10,11].

The disruption of hippocampal cytoarchitecture, altered neuron–glia interactions, and the establishment of a chronic pro-inflammatory environment during prenatal and early postnatal development substantially increase susceptibility to neuropsychiatric and neurodegenerative disorders later in life [7]. These conditions include autism spectrum disorder and attention-deficit/hyperactivity disorder during childhood, schizophrenia, depression, and anxiety disorders during adolescence and adulthood, as well as mild cognitive impairment and the clinical onset of Alzheimer’s disease, Parkinson’s disease, and other neuropsychiatry disorders [12]. Comparable long-term consequences have also been demonstrated in experimental animal models. Offspring born to mothers subjected to MIA exhibit persistent behavioral, molecular, and neuropathological abnormalities extending into adulthood [4,5,8,9,10,11]. For instance, wild-type (WT) mice prenatally exposed to polyinosinic–polycytidylic acid [poly(I:C)] exhibited impaired working memory in the Y-maze at 15 months of age, accompanied by elevated plasma IL-1β concentrations and increased hippocampal expression of *Il1a* and *Il6* [5]. These animals also demonstrated an increased number of CD68+ amoeboid microglia and the accumulation of amyloid precursor protein (APP) and its cleavage products, including soluble APP (sAPP), C-terminal fragments (CTFs), and APP intracellular domain (AICD), as well as increased phosphorylation of tau at Thr205 together with a redistribution of phosphorylated tau from axonal to somatodendritic compartments of hippocampal neurons [8]. Similarly, CD-1 mice prenatally exposed to LPS exhibited progressive age-dependent impairments beginning at 12 months of age, with marked deterioration by 18 and 22 months. These animals displayed reduced locomotor performance, impaired balance, decreased exploratory activity, and deficits in object recognition, spatial learning, and memory, as assessed by a battery of behavioral tests including nesting behavior, beam walk, open field, the elevated plus maze, object location recognition, and the radial six-arm water maze [9]. Behavioral deterioration was accompanied by increased anxiety and enhanced immunohistochemical staining for Aβ1-42, phosphorylated tau, and glial fibrillary acidic protein (GFAP) in the hippocampus compared with age-matched controls. Notably, both neuropathological and behavioral abnormalities progressively worsened with aging [9]. Furthermore, C57BL/6J mice prenatally exposed to poly(I:C) on gestational day 17 exhibited increased hippocampal levels of Aβ1-40 at 12 months of age, together with elevated expression of the Alzheimer’s disease-associated genes *App*, β-site amyloid precursor protein-cleaving enzyme 1 (*Bace1*), Anterior Pharynx Defective 1 Homolog B (*Aph1b*), and the Aβ-degrading enzyme Membrane Metallo-Endopeptidase (*Mme*), compared with control animals. In C57BL/6J mice at 12 months of age following prenatal poly(I:C) administration on gestational day 17, hippocampal Aβ (Aβ1–40) content was increased, along with increased expression of *App*, *Bace1*, *Aph1b*, and the Aβ-degrading enzyme *Mme*, compared with controls [10].

The molecular mechanisms underlying MIA-induced neurodegeneration include the dysregulation of APP metabolism accompanied by increased expression of BACE1, activation of pro-inflammatory cytokines, particularly interleukin-18 (IL-18), and impaired expression and synthesis of neurotrophic factors, primarily brain-derived neurotrophic factor (BDNF) [11,13]. These alterations are closely associated with the activation and functional dysregulation of microglia, the resident macrophages and principal immune cells of the CNS. Polarization of microglia toward the pro-inflammatory M1 phenotype together with the development of oxidative stress further accelerates neurodegenerative processes [14,15].

Most experimental studies investigating MIA-induced neurodegeneration have focused primarily on male offspring [10,16,17]. However, increasing evidence suggests that sex differences substantially influence the susceptibility to neurodevelopmental and neurodegenerative disorders following prenatal immune activation. Epidemiological studies indicate that women are considerably more susceptible to stress-related psychiatric disorders and neurodegenerative diseases, with an approximately two-fold higher lifetime risk compared with men [18,19]. Similar sex-dependent differences have been demonstrated in experimental models, where MIA results in more pronounced impairments of spatial memory, increased anxiety-like behavior, and deficits in social interaction in female offspring compared with males [20,21].

Sex-specific cognitive impairment following MIA may reflect differences in the development of neuroinflammation, regulation of neurotrophic signaling, and compensatory neuroplasticity between males and females. In a mouse model of MIA induced by poly(I:C) administration (5 mg/kg) on gestational day 9.5, female offspring exhibited more pronounced alterations in microglial morphology, greater phagolysosomal activity reflected by increased CD68 expression, and higher levels of the integrin CD11b, resulting in enhanced synaptic pruning compared with male offspring [22]. Similarly, in newborn rats prenatally exposed to LPS (100 μg/kg, gestational day 17), female offspring exhibited reduced expression of the neural stem cell marker *Sox2* and the astroglial progenitor marker *Sox9* in the prefrontal cortex. In contrast, male offspring demonstrated increased numbers of microglial cells together with a shift toward the pro-inflammatory M1 phenotype. Notably, this response was accompanied by enhanced expression of *Hif1α*, suggesting the activation of endogenous neuroprotective mechanisms that may partially compensate for inflammatory damage [23]. Understanding these sex-dependent mechanisms is essential for the development of personalized approaches to the early diagnosis and treatment of MIA-associated neuropsychiatric and neurodegenerative disorders.

Therefore, investigating the long-term consequences of MIA with consideration of biological sex is of considerable scientific and clinical importance. Such studies may facilitate the identification of novel biomarkers and pathogenetic mechanisms underlying neurodegeneration, ultimately improving strategies for the prevention and treatment of cognitive impairment. Accordingly, the primary objective of the present study was to characterize the long-term effects of MIA induced by prenatal LPS exposure on neuronal and microglial phenotypes in the offspring depending on biological sex. The primary outcomes included key behavioral parameters as well as neuronal and microglial morphological characteristics. Secondary outcomes included the expression of genes involved in pro-inflammatory environment and microglial activation, amyloid- and tau-associated pathways, neurotrophic signaling and neuroprotection, and neuronal differentiation. These molecular analyses were intended to provide mechanistic context for the cellular and behavioral phenotypes associated with prenatal MIA exposure. In addition, exploratory analyses were performed to identify sex-specific molecular signatures and potential associations among behavioral, cellular, and molecular alterations following prenatal MIA exposure. These analyses were considered hypothesis-generating and were not intended to establish individual molecular markers as definitive biomarkers of MIA-associated alterations related to neurodegeneration.

## 2. Results

### 2.1. MWM Test Revealed Sex Differences in Search Strategy in Prenatally LPS-Treated Offspring

**Figure 1:**
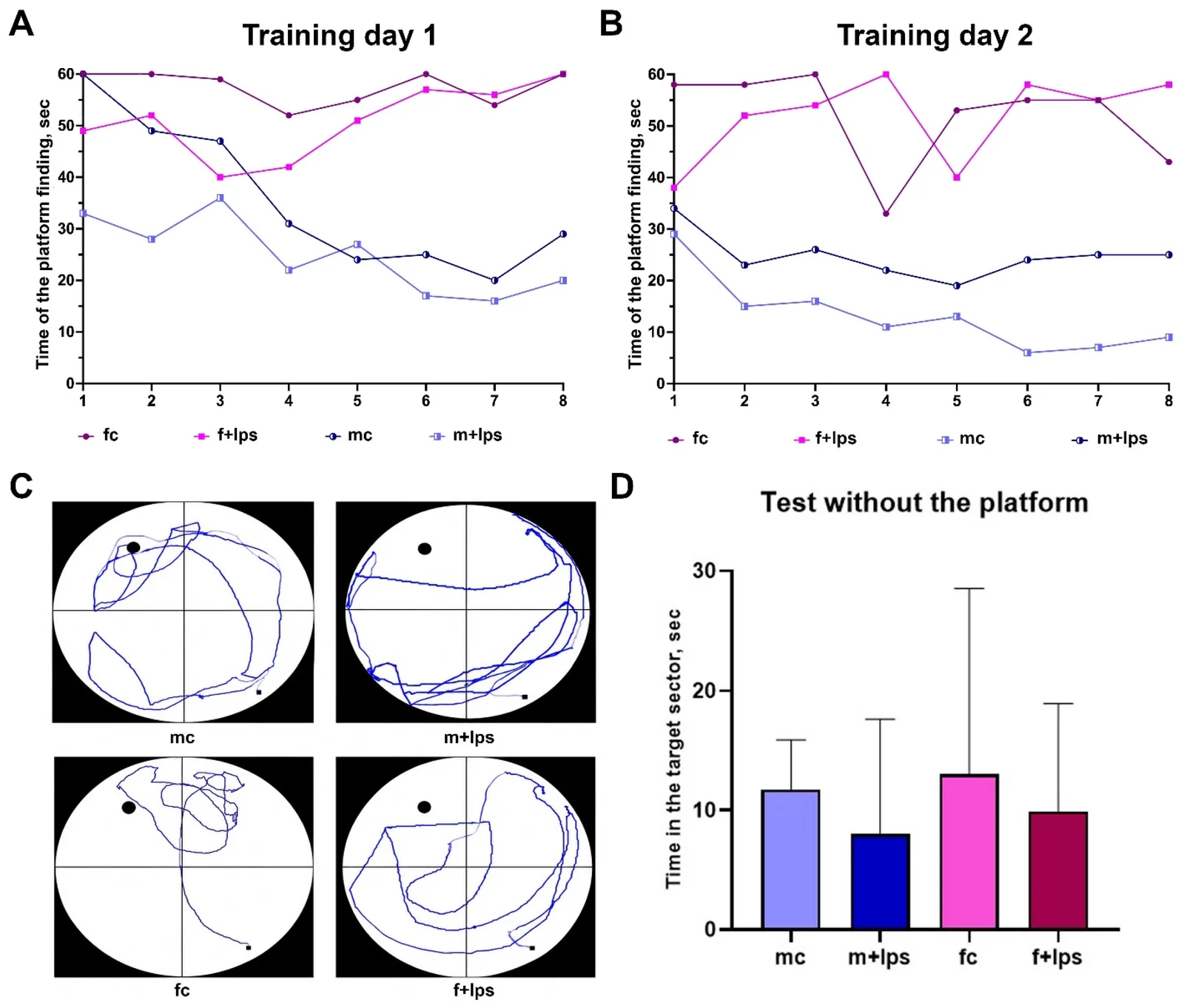
(**A**,**B**)—Time required to locate the hidden platform during MWM acquisition trials on Day 1 (**A**) and Day 2 (**B**) in 12-month-old control and prenatally LPS-treated Wistar rats. (**C**)—Representative swimming trajectories during the probe trial performed six days after training. (**D**)—Time (s) spent in the target quadrant during the probe trial. mc—control males, m+lps—prenatally LPS-treated males, fc—control females, f+lps—prenatally LPS-treated females (*n* = 7 per group). Multiple comparisons: Scheirer–Ray–Hare test followed by Dunn’s post hoc test with Benjamini–Hochberg correction. Data presented as Me (LQ 25%; UQ 75%).

An assessment of spatial learning and memory using the MWM demonstrated sex differences in behavioral performance. Females from both the control and prenatally LPS-treated groups required significantly more time to locate the hidden platform than males of the corresponding groups during the acquisition trials (Figure 1A,B). During the probe trial performed six days after training, when the platform had been removed, no significant differences were observed among the experimental groups in the time spent within the target quadrant (Figure 1D). However, researcher observatory analysis of the swimming trajectories revealed distinct behavioral strategies. Most prenatally LPS-treated females exhibited markedly reduced exploratory activity and predominantly passive drifting behavior, resulting in incidental rather than goal-directed entry into the target quadrant (Figure 1C).

### 2.2. EPM Showed Increased Anxiety in Both Sexes of Prenatally LPS-Treated Offspring

**Figure 2:**
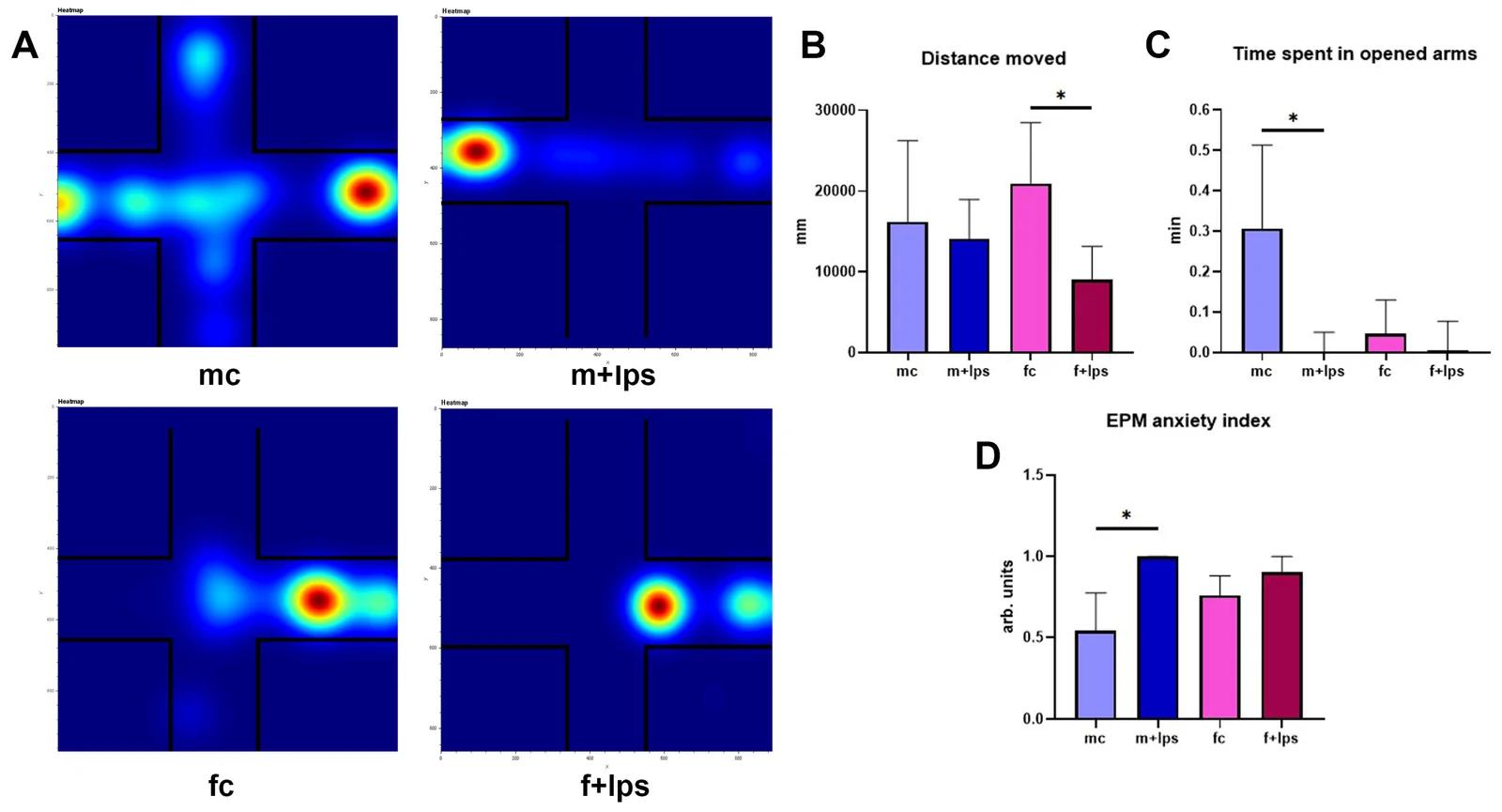
(**A**)—Representative heat maps of movement trajectories in the EPM in 12-month-old control and prenatally LPS-treated Wistar rats. mc—control males: active movement between opened and closed arms, m+lps—prenatally LPS-treated males: predominant sitting in closed arms, fc—control females: moderate movement between the center and closed arms, f+lps—prenatally LPS-treated females: predominant sitting in closed arms; (**B**)—The total average distance traveled in the EPM; (**C**)—Time spent in opened arms of EPM test; (**D**)—Anxiety index calculated based on data from EPM test. *n* = 7 per group. *—*p* < 0.05. Multiple comparisons: Scheirer–Ray–Hare test followed by Dunn’s post hoc test with Benjamini–Hochberg correction. Data presented as Me (LQ 25%; UQ 75%).

Behavioral assessment in the EPM demonstrated increased anxiety-like behavior in both male and female offspring prenatally exposed to LPS. Compared with respective control groups, the experimental females exhibited reduced locomotor activity, reflected by a shorter total distance traveled, whereas the males spent less time in the open arms of the maze (Figure 2A–C). Notably, although both sexes altered behavior, the anxiety index was significantly higher in prenatally LPS-treated males relative to control males (H = 6.296, *p* = 0.012, η^2^H = 0.252) (Figure 2D), whereas the main effect of sex was not significant (H = 0.003, *p* = 0.953, η^2^H = 0). The sex × LPS interaction effect was pronounced, but did not reach statistical significance (H = 2.888, *p* = 0.089, η^2^H = 0.073).

### 2.3. The Percentage of Hyperchromic Pyknotic Neurons Was Elevated Predominantly in Prenatally LPS-Treated Females in the Hippocampus

**Figure 3:**
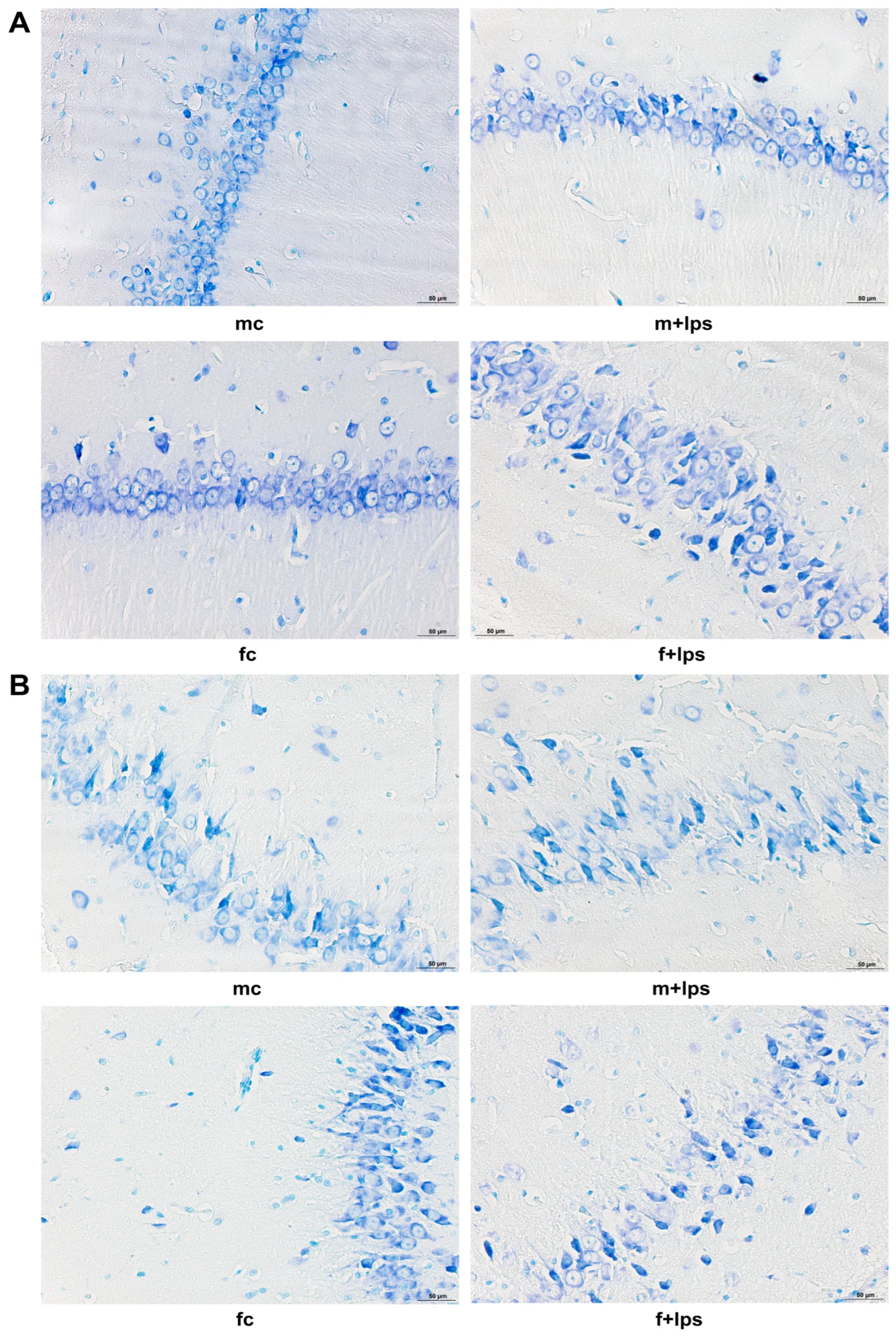
(**A**)—Hyperchromic neurons in the CA1 zone of the hippocampus in 12-month-old control and prenatally LPS-treated Wistar rats; (**B**)—Hyperchromic neurons in the CA3 zone of the hippocampus in 12-month-old control and prenatally LPS-treated Wistar rats. mc—control males, m+lps—prenatally LPS-treated males, fc—control females, f+lps—prenatally LPS-treated females (*n* = 7 per group). Nissl staining, ×400.

**Figure 4:**
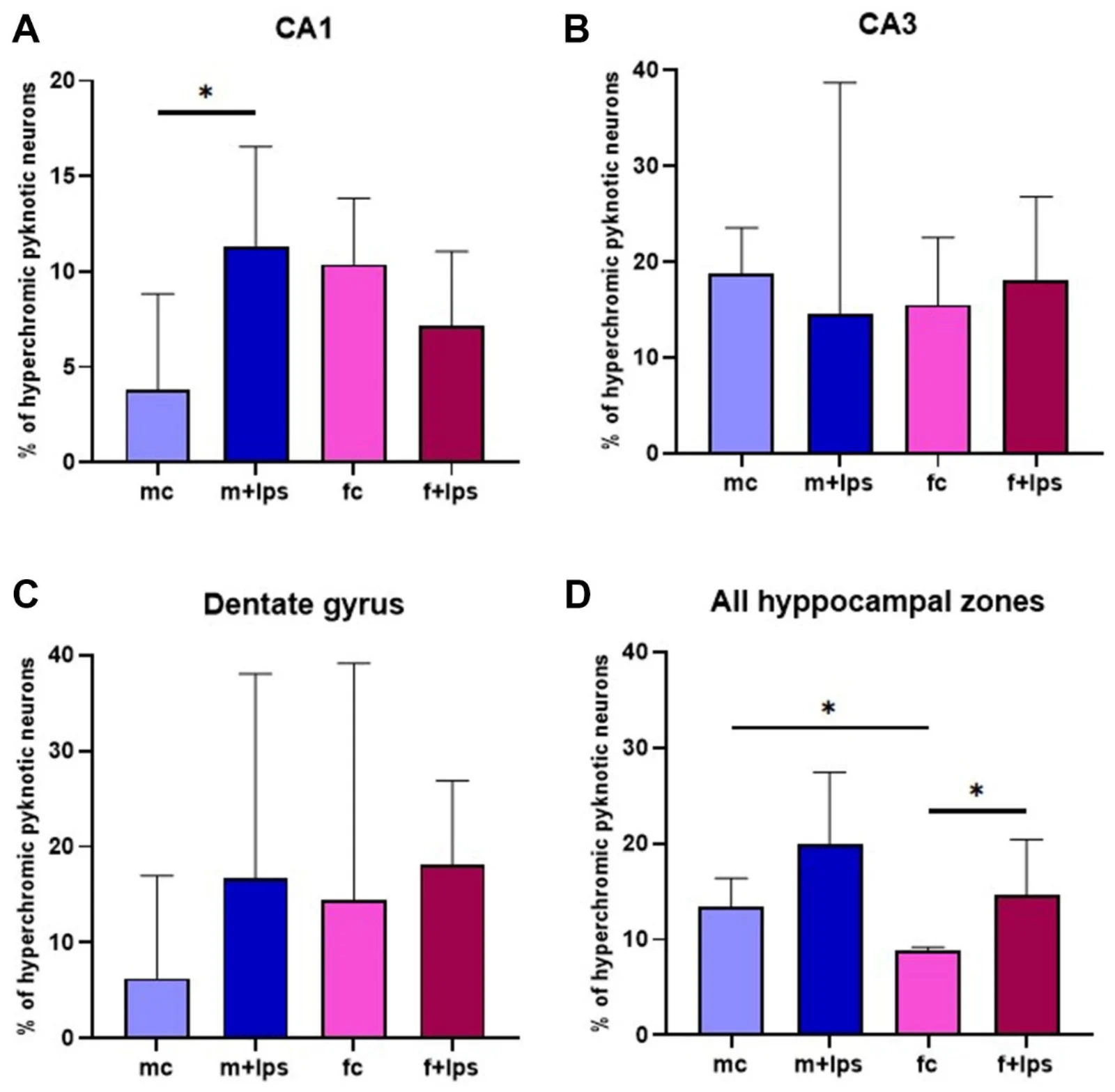
Percentage of hyperchromic pyknotic neurons in the CA1 region (**A**), CA3 region (**B**), dentate gyrus (**C**), and overall across all hippocampal subregions (**D**) in 12-month-old control and prenatally LPS-treated Wistar rats. mc—control males, m+lps—prenatally LPS-treated males, fc—control females, f+lps—prenatally LPS-treated females (*n* = 7 per group). *—*p* <0.05. Multiple comparisons: Scheirer–Ray–Hare test followed by Dunn’s post hoc test with Benjamini–Hochberg correction. Data presented as Me (LQ 25%; UQ 75%).

Nissl staining of the hippocampus revealed hyperchromic pyknotic neurons in all groups. These neurons were characterized by a shrunken polygonal shape, intense cytoplasmic staining, and the absence of a discernible nucleus, consistent with markedly reduced functional activity (Figure 3). Morphometric analysis of individual hippocampal subregions (CA1, CA3, dentate gyrus) compared with the corresponding control group showed a higher relative number of pyknotic hyperchromic neurons in the CA1 zone only in prenatally LPS-treated males (Figure 4A). No significant differences were detected in the CA3 region or dentate gyrus between control and experimental groups of either sex (Figure 4B,C). However, when all hippocampal subregions were analyzed collectively, prenatally LPS-treated females showed a significantly greater overall proportion of hyperchromic pyknotic neurons than control females (H = 4.70, *p* = 0.030, η^2^H = 0.137) (Figure 4D). In addition, sex differences were observed within the control groups, with females exhibiting a lower percentage of hyperchromic neurons than males (H = 4.05, *p* = 0.044, η^2^H = 0.113), whereas the sex × LPS interaction was not significant (H = 2.07, *p* = 0.150) (Figure 4D).

### 2.4. Morphological Features of Microglia and Astrocytes Were Altered in Both Sexes of Prenatally LPS-Treated Offspring

**Figure 5:**
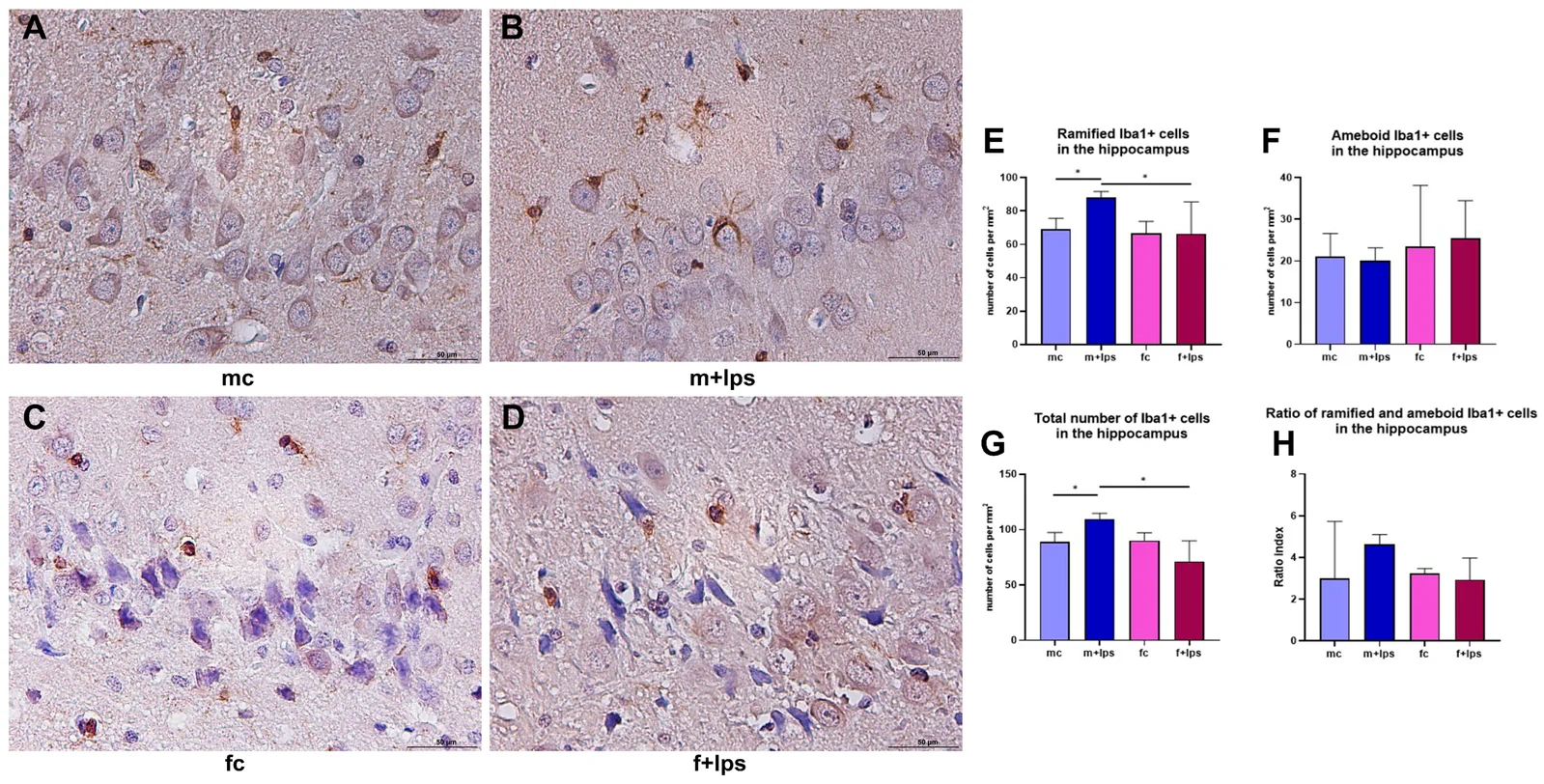
Morphological characteristics and quantitative analysis of Iba1+ microglia in the hippocampus of 12-month-old control and prenatally LPS-treated Wistar rats. (**A**) (mc)—control males: predominantly ramified microglia (approximately 15–30 μm) with thin, moderately branched processes. (**B**) (m+lps)—prenatally LPS-treated males: predominantly ramified microglia with morphology similar to that of controls. (**C**) (fc)—control females: enlarged (>30 μm), spheroid, swollen, hypertrophic microglia. (**D**) (f+lps)—prenatally LPS-treated females: enlarged spheroid microglia with hypertrophic morphology. Iba1 primary antibody + HRP-conjugated secondary antibody and hematoxylin staining. The scale bar represented 20 μm. (**E**)—Number of ramified Iba1+ microglia; (**F**)—Number of amoeboid Iba1+ microglia; (**G**)—Total number of Iba1+ microglial cells; (**H**)—Ramified-to-amoeboid microglia ratio. mc—control males, m+lps—prenatally LPS-treated males, fc—control females, f+lps—prenatally LPS-treated females (*n* = 7 per group). *—*p* < 0.05. Multiple comparisons: Scheirer–Ray–Hare test followed by Dunn’s post hoc test with Benjamini–Hochberg correction. Data presented as Me (LQ 25%; UQ 75%).

IHC revealed sex-dependent alterations in the morphology of Iba1+ microglia following prenatal LPS exposure. In both control and prenatally LPS-treated males, microglia predominantly exhibited a ramified morphology (Figure 5A,B,E). As previously described, such morphology is characteristic of the homeostatic state [12]. Compared with both control males and prenatally LPS-treated females, prenatally LPS-treated males demonstrated a significantly greater number of ramified Iba1+ microglial cells (H = 3.84, *p* = 0.0495, η^2^H = 0.134), whereas there was no significant sex × LPS interaction (H = 0.92, *p* = 0.337, η^2^H = 0) (Figure 5A,B,E). Prenatally LPS-treated females exhibited a trend to lower ramified-to-amoeboid microglia ratio than prenatally LPS-treated males, indicating a shift toward a more reactive microglial phenotype; however, it did not reach statistical significance (Figure 5C,D,H). The total number of Iba1+ microglial cells in the hippocampus was significantly increased in prenatally LPS-treated males compared with control males (H = 3.84, *p* = 0.0495, η^2^ = 0.147), whereas no significant difference was observed between female control and LPS-treated females despite also having a mediumsize effect (H = 2.81, *p* = 0.093, η^2^ = 0.108) (Figure 5G). A trend toward a sex × LPS interaction was observed (H = 3.29, *p* = 0.070, η^2^ = 0.127), suggesting a potentially sex-dependent response to prenatal LPS exposure. Nevertheless, the total number of Iba1+ microglia was significantly higher in prenatally LPS-treated males than in both control males and prenatally LPS-treated females according to Dunn’s post hoc test (Figure 5G).

**Figure 6:**
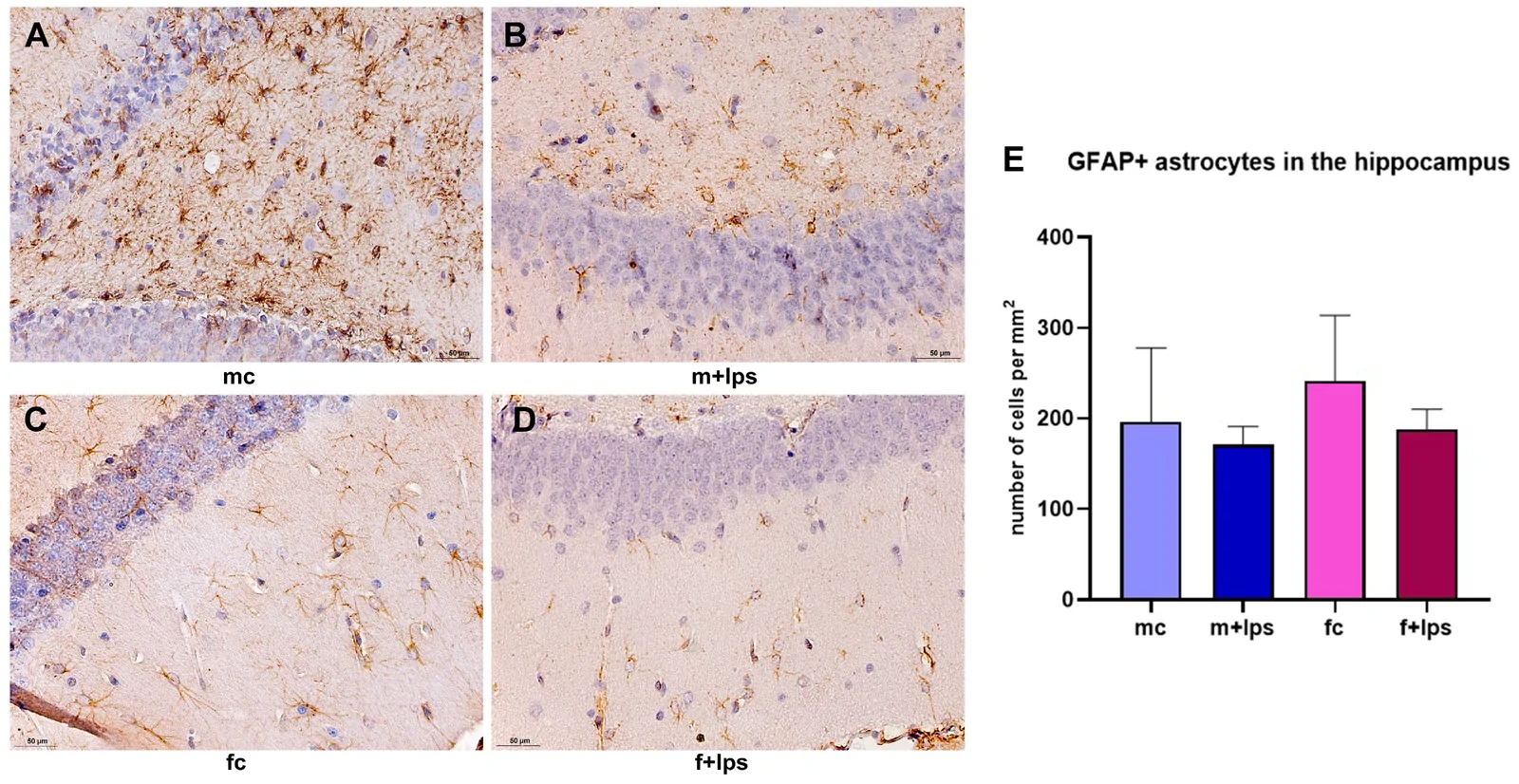
GFAP+ astrocytes in the hippocampus of 12-month-old control and prenatally LPS-treated Wistar rats. (**A**) (mc)—control males: numerous small GFAP+ astrocytes with branched processes; (**B**) (m+lps)—prenatally LPS-treated males: few small GFAP+ astrocytes with thin, fragmented processes; (**C**) (fc)—control females: few small GFAP+ astrocytes with thin, sparse processes; (**D**) (f+lps)—prenatally LPS-treated females: few small GFAP+ astrocytes with thin, fragmented processes. GFAP primary antibody + HRP-conjugated secondary antibody and hematoxylin staining. The scale bar represented 20 μm. (**E**)—number of GFAP+ astrocytes in the hippocampus of 12-month-old control and prenatally LPS-treated Wistar rats. mc—control males, m+lps—prenatally LPS-treated males, fc—control females, f+lps—prenatally LPS-treated females (*n* = 7 per group). Multiple comparisons: Scheirer–Ray–Hare test followed by Dunn’s post hoc test with Benjamini–Hochberg correction. Data presented as Me (LQ 25%; UQ 75%).

IHC staining for GFAP revealed substantial interindividual variability in both the morphology and abundance of GFAP+ astrocytes among animals (Figure 6A–D). However, quantitative morphometric analysis demonstrated no significant differences in the number of GFAP+ astrocytes between control and prenatally LPS-treated animals of either sex (Figure 6E).

### 2.5. Prenatal LPS Exposure Induced Sex-Independent Upregulation of Inflammatory and Senescence-Associated Genes in the Hippocampus

**Figure 7:**
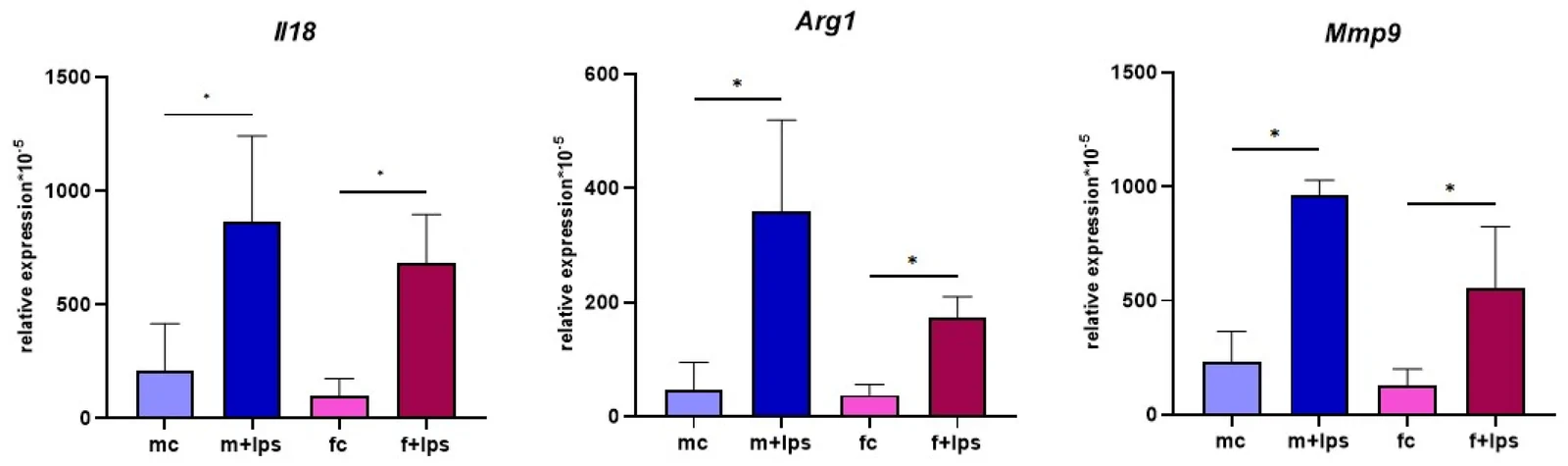
Relative mRNA expression levels of *Il18*, *Arg1*, and *Mmp9* in the hippocampus of 12-month-old control and prenatally LPS-treated Wistar rats. mc—control males, m+lps—prenatally LPS-treated males, fc—control females, f+lps—prenatally LPS-treated females (*n* = 7 per group). *—*p* < 0.05. Multiple comparisons: Scheirer–Ray–Hare test followed by Dunn’s post hoc test with Benjamini–Hochberg correction. Data presented as Me (LQ 25%; UQ 75%).

Both male and female offspring prenatally exposed to LPS exhibited significantly increased hippocampal expression of the pro-inflammatory cytokine *Il18*, indicating the establishment of a persistent pro-inflammatory environment (Figure 7). Similarly, the expression of *Arg1*, a marker associated with the anti-inflammatory M2 phenotype, and *Mmp9*, a marker of cellular senescence and extracellular matrix remodeling, was significantly elevated in both sexes following prenatal LPS exposure (Figure 7). No sex differences in the expression levels of *Il18*, *Arg1*, or *Mmp9* were detected in either the control or experimental groups.

**Figure 8:**
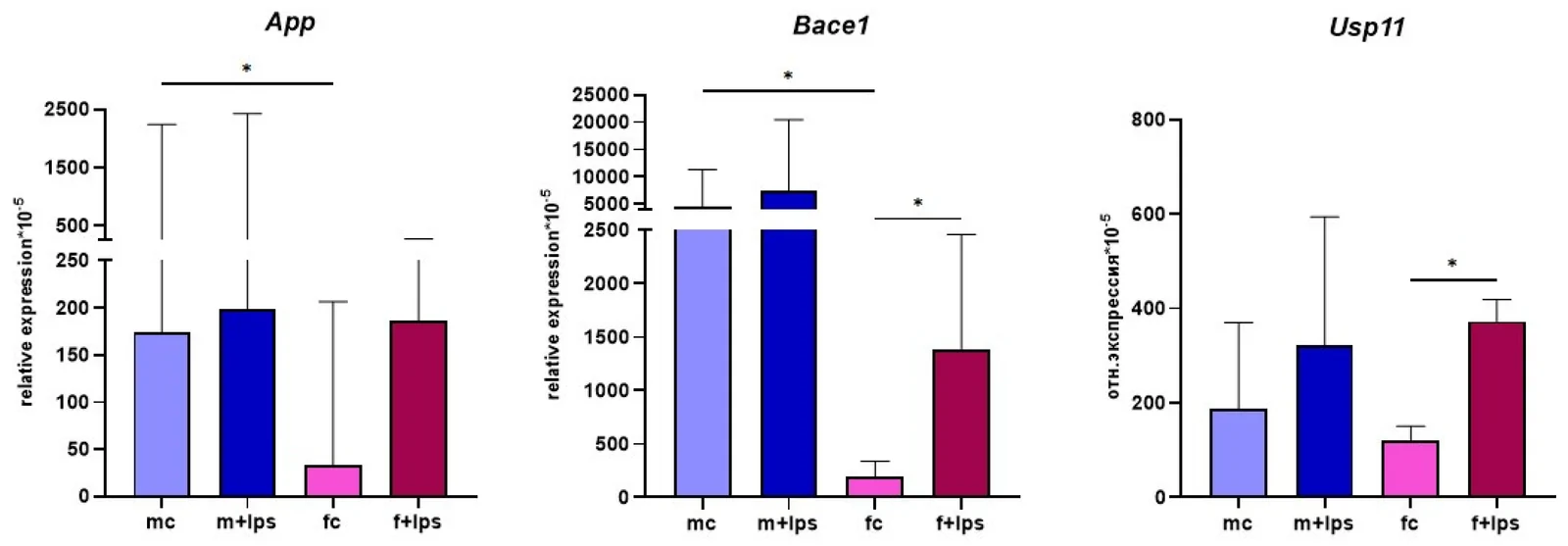
Relative mRNA expression levels of *App*, *Bace1*, and *Usp11* in the hippocampus of 12-month-old control and prenatally LPS-treated Wistar rats. mc—control males, m+lps—prenatally LPS-treated males, fc—control females, f+lps—prenatally LPS-treated females (*n* = 7 per group). *—*p* < 0.05. Multiple comparisons: Scheirer–Ray–Hare test followed by Dunn’s post hoc test with Benjamini–Hochberg correction. Data presented as Me (LQ 25%; UQ 75%).

Sex differences in the expression of genes associated with amyloid metabolism were observed in the hippocampus (Figure 8). Under control conditions, female rats exhibited significantly lower *App* mRNA expression than males. Likewise, hippocampal expression of *Bace1* was lower in control females than in control males, which may be attributable to the lower basal expression of *App*. Notably, prenatal LPS exposure significantly increased *Bace1* expression exclusively in female offspring, whereas no significant changes were detected in males (Figure 8).

The deubiquitinating enzyme *Usp11*, which has been implicated in neuroinflammation and neurodegenerative disorders, displayed a similar expression pattern. By stabilizing specific proteins through deubiquitination, USP11 frequently promotes microglial activation, astrogliosis, and neuronal apoptosis [24]. Although no sex differences were observed in the control groups, hippocampal Usp11 expression was only significantly upregulated in prenatally LPS-treated females compared with their corresponding controls (Figure 8).

**Figure 9:**
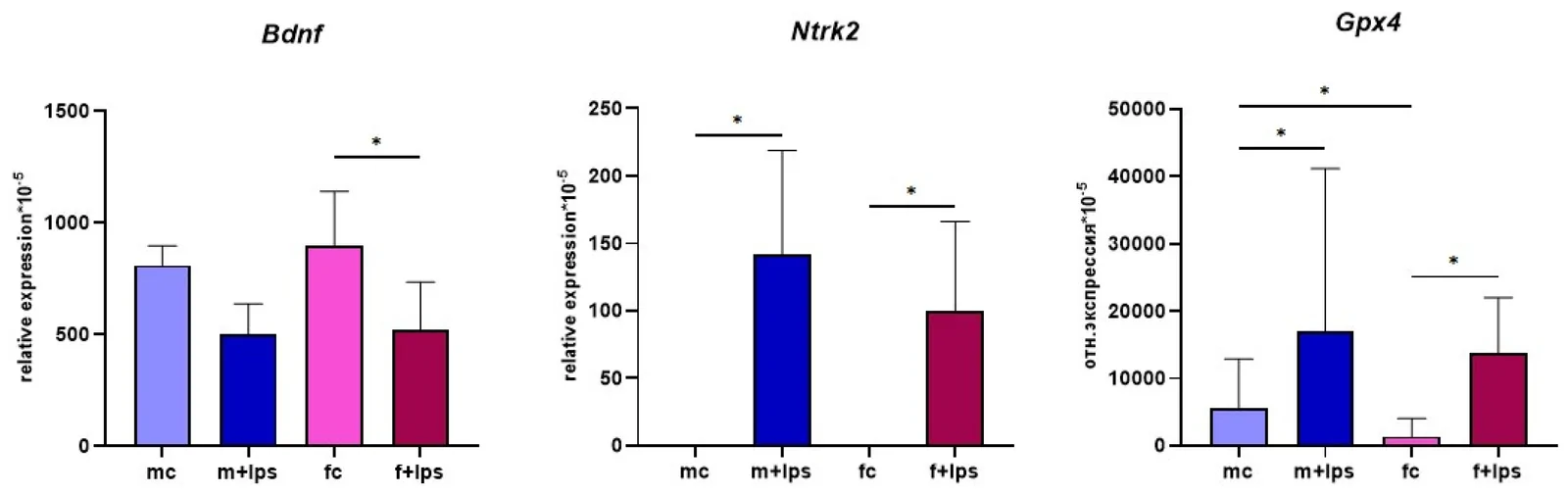
Relative mRNA expression levels of *Bdnf*, *Ntrk2*, and *Gpx4* in the hippocampus of 12-month-old control and prenatally LPS-treated Wistar rats. mc—control males, m+lps—prenatally LPS-treated males, fc—control females, f+lps—prenatally LPS-treated females (*n* = 7 per group). *—*p* < 0.05. Multiple comparisons: Scheirer–Ray–Hare test followed by Dunn’s post hoc test with Benjamini–Hochberg correction. Data presented as Me (LQ 25%; UQ 75%).

A significant reduction in hippocampal *Bdnf* expression was observed exclusively in prenatally LPS-treated females (Figure 9). In contrast, the expression of *Ntrk2*, encoding the high-affinity receptor for BDNF (TrkB), was significantly increased in both male and female offspring following prenatal LPS exposure, suggesting the activation of a compensatory mechanism in response to impaired neurotrophic signaling (Figure 9). Similarly, hippocampal *Gpx4* expression was significantly elevated in both sexes compared with their respective control groups, indicating enhanced antioxidant defense in response to persistent pro-inflammatory background (Figure 9). Under physiological conditions, female control animals exhibited lower *Gpx4* expression than control males, demonstrating a sex difference in the basal antioxidant status of the hippocampus.

**Figure 10:**
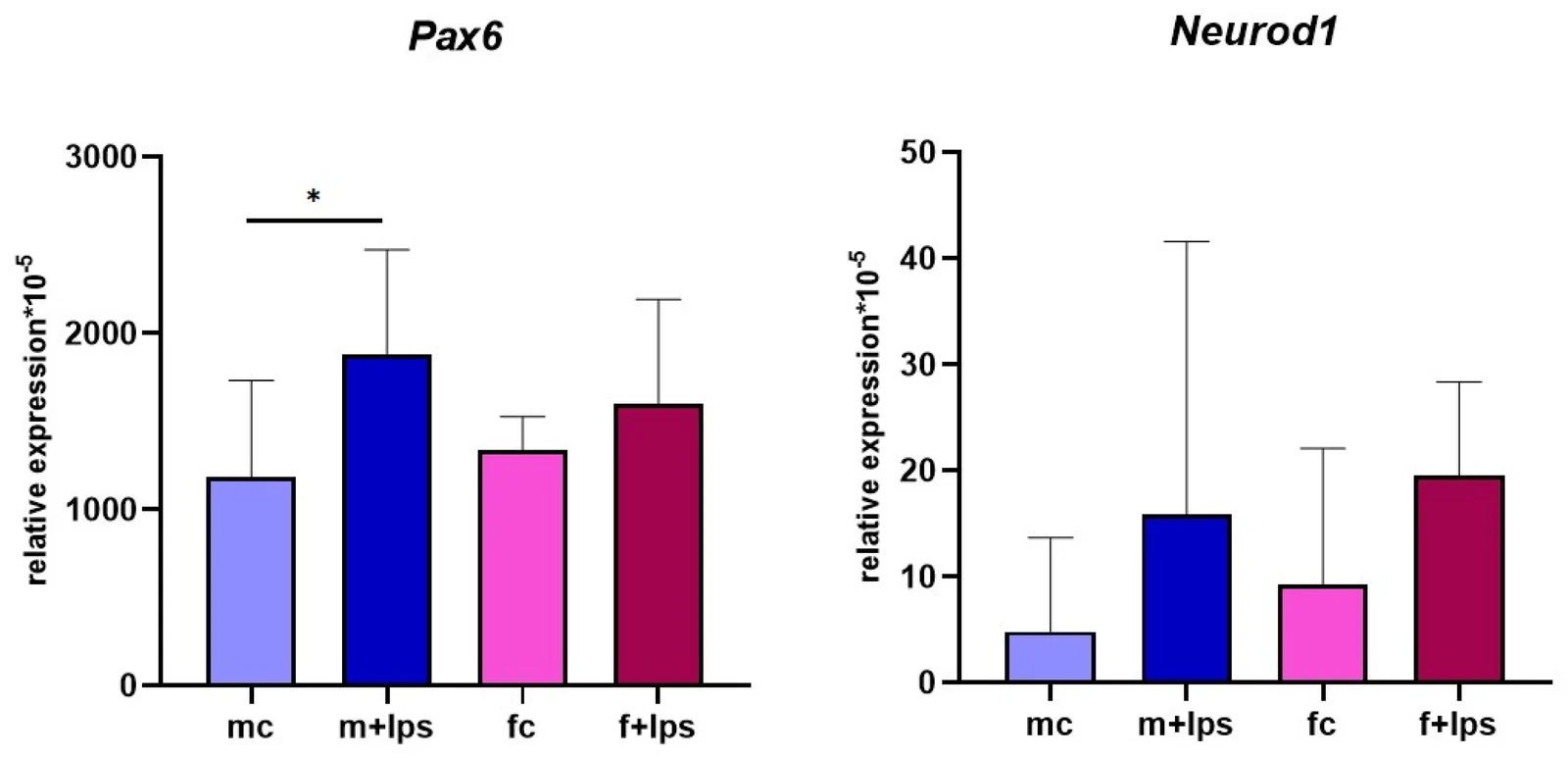
Relative mRNA expression levels of *Pax6* and *Neurod1* in the hippocampus of 12-month-old control and prenatally LPS-treated Wistar rats. mc—control males, m+lps—prenatally LPS-treated males, fc—control females, f+lps—prenatally LPS-treated females (*n* = 7 per group). *—*p* < 0.05. Multiple comparisons: Scheirer–Ray–Hare test followed by Dunn’s post hoc test with Benjamini–Hochberg correction. Data presented as Me (LQ 25%; UQ 75%).

Among the genes associated with neurogenesis, hippocampal *Pax6* expression was only significantly increased in prenatally LPS-treated males (Figure 10). In contrast, *Neurod1* expression remained unchanged in both male and female offspring following prenatal LPS exposure (Figure 10).

Thus, in the long-term period following MIA exposure, changes in behavior and in the morphofunctional state of neurons and microglia in the hippocampus are observed in both males and females. The findings are summarized in Table 1.

## 3. Discussion

The present study demonstrates that MIA induces long-lasting, sex-dependent behavioral, morphological, and molecular alterations in the offspring that persist until at least 12 months of age. Although both male and female offspring exhibited greater vulnerability to the long-term consequences of prenatal immune activation than their respective controls, the nature and severity of these alterations differed between the sexes.

In both the control and prenatally LPS-treated groups, female rats required more time to locate the hidden platform in the Morris water maze than males, indicating slower acquisition of the spatial task. However, cognitive impairment was considerably more pronounced in prenatally LPS-treated females, who predominantly exhibited passive behavior and an absence of goal-directed exploration during both the acquisition and probe trials. These findings are consistent with well-established sex differences in behavioral responses to stress. Compared with males, females generally adopt more passive or avoidance-oriented coping strategies, emphasizing caution and social support rather than active exploration [25,26,27]. Such behavioral differences have been associated with greater susceptibility of the female brain to adverse prenatal and postnatal influences and may contribute to the more severe behavioral phenotype observed in females following prenatal immune activation [28]. The reduced exploratory behavior displayed by 12-month-old prenatally LPS-treated females during the probe trial may also reflect the impairment of hippocampus-dependent spatial memory retrieval [29]. The hippocampus plays a central role in the encoding and retrieval of spatial information, and the disruption of hippocampal function is known to compromise goal-directed navigation. Our findings are consistent with those of Chlodzinska et al. (2011), who reported that female offspring prenatally exposed to LPS performed significantly worse than controls in the nest-building test, which reflects the functional integrity of the hippocampus [30].

Although female offspring exhibited a more pronounced reduction in locomotor activity in the EPM, male offspring displayed anxiety-like phenotype, characterized by fewer entries into the open arms and a significantly higher anxiety index. Similar sex-dependent behavioral alterations following MIA have been reported previously. At postnatal day 61, female offspring exposed to MIA exhibited hyperactivity and impaired spatial working memory, reflected by fewer entries into the novel arm of the Y-maze, whereas male offspring displayed reduced locomotor activity in the same test [20]. Likewise, prenatal poly(I:C) exposure impaired social preference and associative memory in C57BL/6 mouse offspring of both sexes at postnatal day 80, whereas deficits in spatial working memory were observed exclusively in females [21]. Furthermore, Wellman et al. (2018) demonstrated that female offspring exposed to prenatal MIA exhibited long-term object-recognition memory deficits in the Novel Object Recognition Test (NORT) with preserved short-term memory [31]. Collectively, these findings indicate that prenatal immune activation differentially affects cognitive and emotional domains in male and female offspring.

Morphometric analysis revealed an increase in pyknotic hyperchromic neurons in the hippocampus of prenatally LPS-treated females when data from the CA1, CA3, and dentate gyrus were analyzed collectively. In contrast, male offspring exhibited a significant increase only in the CA1 region, indicating region-specific hippocampal vulnerability to prenatal immune activation. Hyperchromic neurons are considered early indicators of ischemic and metabolic stress, and their increased abundance may reflect impaired neuronal energy metabolism [32]. Neuronal dysfunction disrupts the balance between excitatory and inhibitory neurotransmission, alters neuronal network activity, impairs learning, and reduces synaptic plasticity [32]. Consistent with our findings, Hao et al. (2010) demonstrated that prenatal LPS exposure resulted in neuronal loss, decreased synaptophysin level, and increased GFAP level in the hippocampal CA1 region of offspring of both sexes, with these alterations becoming progressively more pronounced between 3 and 20 months of age [3]. Furthermore, transient inactivation of the CA1 region with lidocaine during Novel Object Preference (NOP) and MWM training impaired both object recognition and spatial navigation in C57BL/6J mice [33], highlighting the pivotal role of this hippocampal subregion in learning and memory and supporting the functional significance of our morphological findings.

IHC analysis revealed sex differences in microglial morphology following prenatal immune activation. Males from both the control and experimental groups retained predominantly ramified microglia, which are characteristic of the homeostatic state [12,34]. According to Vinet et al. (2012), ramified microglia continuously survey the brain microenvironment and protect hippocampal neurons under both physiological and pathological conditions [34]. In addition, these cells regulate complement-mediated synaptic pruning through signaling pathways involving complement proteins C1q and C3 and the microglial receptor CR3, thereby contributing to normal cognitive function [34,35,36]. Importantly, ramified microglia may retain phagocytic activity without undergoing classical pro-inflammatory polarization, representing a neuroprotective phenotype compatible with preserved cognitive function. This concept is further supported by the recent study of Gallo et al. (2026), who demonstrated, in both primary mouse microglial cultures and transgenic Tg2576 mice, that the synthetic sulfolipid Sulfavant A binds Triggering Receptor Expressed on Myeloid Cells 2 (TREM2), enhancing microglial phagocytosis of β-amyloid without inducing classical pro-inflammatory activation [36]. Therefore, the predominance of ramified microglia observed in prenatally LPS-treated males could be associated with a more preserved spatial memory observed in male offspring.

The persistent upregulation of *Il18* and *Mmp9* observed in both male and female offspring indicates the formation of a pro-inflammatory background and a senescent cell phenotype [37]. In human SH-SY5Y neuroblastoma cells, increased IL-18 production has been associated with elevated Aβ levels, enhanced expression of the amyloidogenic enzymes BACE1 and presenilin-1 (PS-1), activation of caspase-1-dependent pyroptosis [11], and increased oxidative stress through the induction of the antioxidant proteins PRX3–6 (peroxiredoxins) and biliverdin reductase A (BLVRA) [38]. These processes may, in turn, stimulate the expression of matrix metalloproteinases, including MMP2 and MMP9 [39,40]. Elevated hippocampal IL-18 expression has also been linked to depressive-like behavior and memory impairment in the offspring of Sprague–Dawley rats exposed to prenatal stress [41]. MMP9 contributes to extracellular matrix degradation, disruption of blood–brain barrier integrity, infiltration of peripheral immune cells into the CNS, activation of microglia, and remodeling of synaptic plasticity [42,43]. Microglia produce MMP9 in the CNS under conditions of an established pro-inflammatory background [44]. Furthermore, MMP9 promotes polarization of microglia toward a pro-inflammatory phenotype by enhancing IL-1β signaling, thereby amplifying neuroinflammation and contributing to the development of behavioral abnormalities [45]. Collectively, these findings support the hypothesis that the simultaneous upregulation of *Il18* and *Mmp9* observed in our study reflects the establishment of a self-sustaining pro-inflammatory cascade that may underlie the long-term behavioral deficits induced by MIA. Interestingly, prenatal LPS exposure also increased *Arg1* expression in both sexes. Arginase-1 is widely recognized as a marker of alternatively activated (M2-like) microglia. Therefore, the concurrent upregulation of *Arg1*, *Il18*, and *Mmp9* might potentially reflect activation of compensatory anti-inflammatory mechanisms rather than prerequisite for polarization toward either a pro-inflammatory or an anti-inflammatory phenotype. Mixed microglial activation states have been described during the early stages of neurodegenerative disorders, when protective and detrimental immune responses coexist before overt clinical manifestations become apparent [46]. Despite these common inflammatory responses, female offspring exhibited more pronounced microglial activation at the morphological level. The lower number of ramified microglia observed in prenatally LPS-treated females might partially explain why female offspring displayed more pronounced neuronal alterations and cognitive impairment than males.

RT-qPCR analysis further demonstrated sex-dependent alterations in the hippocampal expression of genes involved in neuroplasticity, pro-inflammatory reactions, and neurodegeneration. The marked increase in *Bace1* expression observed exclusively in female offspring, despite relatively low *App* expression, suggests that this response might be driven primarily by chronic oxidative stress associated with a persistent pro-inflammatory background rather than by enhanced amyloid metabolism itself [47]. In addition to its well-established role in amyloidogenic APP processing, BACE1 regulates synaptic vesicle dynamics and neurotransmitter release [48]. Consequently, moderate increases in BACE1 expression may initially represent an adaptive response aimed at preserving synaptic transmission under inflammatory conditions. Nevertheless, prolonged elevation of BACE1 expression is likely to increase the proportion of APP processed through the amyloidogenic pathway. Increased BACE1 activity has been detected in both the brain tissue and cerebrospinal fluid of patients with mild cognitive impairment during the preclinical stages of Alzheimer’s disease, when amyloid deposition remains minimal [13,49]. Furthermore, accumulating evidence indicates that the β-secretase-derived C-terminal APP fragment (β-CTF/C99) contributes to endosomal–lysosomal dysfunction before detectable amyloid plaque formation, supporting the value of BACE1 as an early biomarker of neurodegeneration [50,51,52].

A similarly female-specific pattern was observed for *Usp11*, whose increased hippocampal expression may indicate enhanced inflammatory signaling. Experimental studies using a rat model of intracerebral hemorrhage (ICH) demonstrated that USP11 amplifies neuroinflammation through the USP11/p53/KLF2 (Kruppel-like Factor 2)/NF-κB signaling pathway, promoting microglial activation, neutrophil infiltration, neuronal apoptosis, and the production of pro-inflammatory mediators, including myeloperoxidase, IL-1β, and Tumor necrosis factor (TNF). Conversely, the suppression of Usp11 via a KLF2-carrying lentiviral plasmid blocked the release of pro-inflammatory cytokines after ICH by reducing p53 levels, thereby decreasing neuronal apoptosis, M1-phenotype microglial activation, and the inflammatory response [24].

BDNF plays a pivotal role in maintaining neurogenesis and synaptic plasticity throughout life [53]. During early brain development, BDNF supports the survival, proliferation, and differentiation of neuronal progenitor cells in neurogenic regions, while also regulating neural circuit formation, synaptogenesis, and axonal growth. In the adult brain, BDNF continues to promote synaptic plasticity, hippocampal neurogenesis within the subgranular zone of the dentate gyrus, and neuroprotection [54,55]. The biological effects of BDNF are primarily mediated through its high-affinity receptor, TrkB, which regulates both structural and functional synaptic plasticity underlying learning and memory [56]. The activation of TrkB initiates multiple intracellular signaling pathways, including MAPK/ERK, PI3K/Akt, and phospholipase Cγ (PLCγ), thereby enhancing synaptic transmission, promoting dendritic spine growth, and facilitating long-term potentiation. In addition, BDNF promotes the clustering of AMPA and NMDA receptors into functional nanodomains within dendritic spines, strengthening synaptic connectivity [57]. BDNF also regulates axonal growth and dendritic arborization during neural circuit formation [58] and protects hippocampal GABAergic neurons from the detrimental effects of LPS-induced inflammation [28]. Previous studies have demonstrated that increased *Bdnf* expression in experimental models of MIA is associated with enhanced dendritic spine density, improved neuronal connectivity, and better cognitive performance [59,60], whereas reduced BDNF levels correlate with hippocampal atrophy and progressive memory decline [61]. The upregulation of *Bdnf* in MIA animal models has been directly linked to increased dendritic spine density, dendritic connectivity, and improved cognitive performance [59,60]. Reduced BDNF levels correlate with memory decline and hippocampal atrophy [61]. Therefore, the selective downregulation of *Bdnf* observed in female offspring may potentially represent one of the key molecular mechanisms underlying their greater vulnerability to long-term cognitive impairment following prenatal immune activation. Increased expression of *Ntrk2*, encoding the TrkB receptor, in both sexes could probably be a compensatory response to BDNF-ligand deficiency. Blocking Ntrk2 expression with the competitive ATP-kinase inhibitor 1NM-PP1 in heterozygous Ntrk2^F616A^ mice, who carry a point mutation in the ATP-binding site of *Ntrk2*, did not affect *Bdnf* expression but increased the expression of *Ntrk2fl* (+48.5 ± 11.9%, *p* = 0.001) and *Ntrk2t1* (+30.6 ± 9.5%, *p* = 0.014) in the medial prefrontal cortex, presumably as an adaptation to reduced NTRK2 signaling [62].

The activation of *Gpx4* expression in prenatally LPS-treated animals of both sexes indicates a gene-level response of endogenous antioxidant defense mechanisms due to chronic oxidative stress accompanying a persistent pro-inflammatory environment. These findings could suggest that compensatory antioxidant capacity remains preserved at 12 months of age, although protein-level measurements are warranted. GPX4 suppresses inflammatory signaling by inhibiting arachidonic acid oxidation and preventing activation of the NF-κB pathway [15,63]. Notably, under physiological conditions, female control animals exhibited significantly lower hippocampal *Gpx4* expression than males, consistent with previous reports [64]. Reduced GPX4 activity may increase susceptibility to lipid peroxidation during chronic inflammation, thereby facilitating amyloidogenic APP processing through enhanced BACE1 activity [65]. Consequently, the relatively low basal expression of *Gpx4* in females may partially explain the selective increase in *Bace1* expression observed following prenatal LPS exposure.

Another curious sex-specific finding was the selective upregulation of *Pax6* in male offspring. Because PAX6 is a master regulator of neurogenesis, increased *Pax6* expression may presumably indicate enhanced regenerative capacity and more effective maintenance of neuronal progenitor populations in males. PAX6 coordinates neuronal proliferation, differentiation, and migration throughout CNS development and remains essential for maintaining neurogenic potential in the adult brain [66]. Consistent with this interpretation, age-related reductions in PAX6 expression have been closely associated with impaired neurogenesis and progressive neuronal dysfunction. Tripathi et al. (2012) demonstrated a gradual decline in both the number of PAX6+ cells and PAX6 protein levels in the olfactory bulb, cerebellum, and hippocampus from postnatal day 0 to old age (70 weeks) in mice [67]. The most pronounced decline in PAX6 was found in the dentate gyrus and CA1, CA2, and CA3 regions of the hippocampus, in the mitral and internal plexiform layers of the olfactory bulb, and in the granular and Purkinje-cell layers of the cerebellum [67]. Moreover, experimental overexpression of PAX6 has been shown to enhance astrocyte-to-neuron conversion and promote neuronal regeneration [68]. Therefore, the increased *Pax6* gene expression observed in male offspring may potentially represent an adaptive mechanism that partially counteracts the detrimental consequences of prenatal immune activation. The absence of change in *Neurod1* expression indicates that the terminal differentiation of neuroblasts is not disrupted. NEUROD1 is vital for the survival and maturation of adult-born neurons through reprogramming of chromatin and transcriptional landscapes; it also drives cell-cycle exit in progenitor cells and promotes rapid neuronal maturation of their progeny [69,70]. In contrast, *Neurod1* expression remained unchanged in both sexes. Because NEUROD1 plays an essential role in neuronal maturation by promoting the cell-cycle exit of neural progenitors, chromatin remodeling, and transcriptional reprogramming required for neuronal differentiation [69,70], the absence of changes in *Neurod1* expression suggests that prenatal immune activation primarily affects the maintenance and regenerative capacity of neural progenitor populations rather than the terminal differentiation of newly generated neurons. Thus, in prenatally LPS-treated males, the activation of *Pax6* expression without a change in terminal differentiation via *Neurod1* may reflect more effective compensatory plasticity—an increased reserve of neuronal precursors without disrupted regulation of their maturation.

## 4. Materials and Methods

### 4.1. Animals

Adult male and female Wistar rats were obtained from the “Nursery of Laboratory Animals” (Branch of the Institute of Bioorganic Chemistry, Russian Academy of Sciences, Pushchino, Russia). All animal procedures were performed in accordance with the European Convention for the Protection of Vertebrate Animals Used for Experimental and Other Scientific Purposes (Strasbourg, 1986) and Directive 2010/63/EU of the European Parliament and of the Council on the protection of animals used for scientific purposes. The experimental protocol was approved by the Bioethics Committee of the Petrovsky National Research Center of Surgery (Protocol No. 8, 29 September 2023) and were reported in accordance with the ARRIVE 2.0 guidelines. Before the experiments, animals were acclimatized for 24 h in the animal facility and housed in groups of three per cage (460 × 300 × 160 mm) under a natural light/dark cycle at 19–21 °C and 40–60% relative humidity. Standard laboratory chow and tap water were provided *ad libitum*.

### 4.2. MIA Model Establishment

A total of 28 adult Wistar rats (14 females and 14 males) were used for breeding. Pregnancy lasted 21–23 days. MIA was modeled by LPS administration in late pregnancy, as previously described [23]. Briefly, on gestational day 17, pregnant females received an intraperitoneal injection of Escherichia coli O26:B6 lipopolysaccharide (LPS; Sigma-Aldrich, St. Louis, MO, USA) at a dose of 100 μg/kg (experimental group; *n* = 7), whereas control animals received an equal volume of physiological saline (control group; *n* = 7).

To assess the severity of the inflammatory response induced by MIA modeling, blood samples were collected from the tail vein of dams under Zoletil anesthesia (4 mg/kg; Virbac Santé Animale, Carros, France) after parturition. Serum calprotectin concentrations were determined using a commercial ELISA kit (FineTest, Wuhan, China) [71]. Elevated serum calprotectin levels, confirming systemic inflammatory activation and persistence of a pro-inflammatory state until delivery, were reported in our previous study [23].

The experimental offspring consisted of 12-month-old male and female rats. To eliminate litter effects, one male and one female offspring were randomly selected from each litter. Consequently, each experimental group contained seven independent litter units (*n* = 7). Offspring of both sexes prenatally exposed to LPS were examined at 1 year of age.

Different researchers were involved in the experiment. A.M.K. performed LPS administration, randomly divided the offspring into groups and coded the samples; A.S.L., A.V.S., E.A.M., and G.M.A. performed behavioral, histological, IHC, and molecular studies, while the group assignment of the samples was not known.

### 4.3. Behavioral Tests

Behavioral analyses were performed in 28 rats divided into four experimental groups (mc—control male, fc—control female, m+lps—prenatally LPS-treated male, f+lps—prenatally LPS-treated female; *n* = 7 per group). The experimenter remained blinded to both treatment group and sex throughout behavioral testing. Animals were acclimated to the testing room for 1 h before each experiment. Following each behavioral session, the apparatus was thoroughly cleaned with 75% ethanol to eliminate olfactory cues.

#### 4.3.1. Morris Water Maze Test (MWM)

Spatial learning and memory were assessed using the MWM. The apparatus consisted of a circular gray pool (150 cm in diameter, 60 cm in height) filled with water to a depth of 30 cm maintained at 22 ± 2 °C. A transparent circular escape platform (8 cm in diameter) was submerged 1.5–2 cm below the water surface in the center of one target quadrant. The pool was located in a room containing stable distal visual cues, which remained unchanged throughout the experiment. Testing was performed under natural lighting. Animals underwent one training session per day for two consecutive days. During each session, rats were released into the pool from eight different starting positions located at equal distances from the platform. Animals that reached the platform remained there for 15 s before being transferred to an individual holding cage for 60 s. Rats failing to locate the platform within 60 s were gently guided to the platform and allowed to remain there for 15 s. Escape latency was recorded during each acquisition trial. Long-term spatial memory was evaluated six days after training using a 60 s probe trial, during which the platform was removed. Swimming trajectories and the time spent in the target quadrant were recorded and analyzed using Biobserve Viewer software (version 3.0, RRID:SCR_014337, Bonn, Germany). During training, the time to find the platform was assessed; during the probe trial, the movement trajectory and the time spent in the target sector were recorded.

#### 4.3.2. Elevated Plus Maze Test (EPM)

Anxiety-like behavior was assessed using the EPM. The apparatus, constructed from black acrylic plates, consisted of two opposing open arms (50 × 11 cm^2^), two opposing closed arms (50 × 11 × 43 cm), and a central sector (10 × 10 cm^2^). The maze was elevated 80 cm above the floor in a quiet, dimly illuminated room (100–150 lux). Each rat was placed individually on the central platform facing one of the closed arms and was allowed to freely explore the maze for 5 min. Animal movements were video-recorded and analyzed using ezTrack software (RRID:SCR_021496, New York, NY, USA, https://github.com/DeniseCaiLab/ezTrack, accessed on 17 May 2026). The following parameters were quantified: total distance traveled, the time spent in the center, time spent in the open arms, time spent in the closed arms, the number of entries into the center, the number of entries into open arms, the number of entries into closed arms, and the anxiety index. The anxiety index was calculated as: 1 − {[(time in open arms/total time) + (entries in open arms/total entries)]/2}, as previously described [72].

### 4.4. Tissue Collection and Processing

Animals were euthanized in a CO_2_ chamber. Hippocampal samples were taken from the right hemisphere (6 mm dorsal to bregma) [73] and fixed in IntactRNA for subsequent quantitative real-time polymerase chain reaction (RT-qPCR) analysis. The left hemisphere was fixed in 10% buffered formalin (BioVitrum, Saint-Petersburg, Russia) for 48 h. After fixation, brain tissue was sectioned at the level of the posterior hippocampus (6 mm dorsal to bregma) [73] and transferred in 70% ethanol. The samples were subsequently dehydrated through a graded ethanol series using a Tissue-Tek VIP5 Jr automated tissue processor (Sakura, Tokyo, Japan) and embedded in Histomix paraffin medium. Histological sections (5–7 μm thick) were prepared using a Microm HM340E microtome (Thermo Fisher Scientific, Waltham, MA, USA).

### 4.5. Morphological Study

Brain sections were Nissl-stained. In the CA1, CA3, and dentate gyrus regions of the hippocampus, the absolute number of neurons within a standard field of view (25,000 μm^2^) and the relative number of hyperchromic pyknotic neurons among all neurons were assessed. Images were acquired using a Leica DM 2500 microscope (Leica Microsystems, Wetzlar, Germany) at ×400.

### 4.6. Immunohistochemical Staining

For immunohistochemical analysis (IHC), frontal brain sections (6.0 mm dorsal to bregma, at the level of the hippocampus) were prepared as previously described [74]. Immunohistochemical staining was performed to identify Iba1+ microglia and GFAP+ astrocytes. The following primary antibodies were used: rabbit anti-Iba1 (1:100; PAC288Ra01, Cloud-Clone Corp., Katy, TX, USA) and rabbit anti-GFAP (1:200; PAA068Ra01, Cloud-Clone Corp., USA). Goat anti-rabbit horseradish peroxidase (HRP)-conjugated secondary antibody (1:500; SAA544Rb1, Cloud-Clone Corp., USA) was used for signal detection. Whole-slide images were acquired using a KF-PRO-005 digital slide scanner (KFBIO, Ningbo, China). Morphometric analysis was performed using QuPath software (version 0.6.0) [75]. The total number of Iba1+ microglial cells and GFAP+ astrocytes were manually counted on 3 sections per animal (the mean number from the 3 sections was calculated) in the hippocampus and cerebral cortex and calculated per 1 mm^2^ of tissue area.

### 4.7. Real-Time qPCR

Total RNA was extracted from hippocampal tissue using ExtractRNA reagent (Evrogen, Moscow, Russia). Complementary DNA (cDNA) was synthesized using the MMLV RT Kit (Evrogen, Russia), and quantitative real-time PCR (RT-qPCR) was performed using qPCRmix-HS SYBR (Evrogen, Russia) containing the fluorescent intercalating dye SYBR Green I on a DTprime Real-Time PCR System (DNA-Technology, Protvino, Russia). The mRNA expression levels of genes encoding *Il18*, *App*, *Bace1*, *Bdnf*, ubiquitin-specific peptidase 11 (*Usp11*), neurotrophic receptor tyrosine kinase 2 (*Ntrk2*), glutathione peroxidase 4 (*Gpx4*), matrix metalloproteinase 9 (*Mmp9*), paired box protein 6 (*Pax6*), neuronal differentiation 1 (*Neurod1*), and arginase 1 (*Arg1*) were quantified in hippocampal samples. The housekeeping gene *Gapdh* was used as reference because it has been demonstrated to be a reliable reference gene for rat brain tissue in both sexes [76]. Relative gene expression was determined using the threshold cycle (Ct) method according to the mathematical model proposed by Pfaffl [77]. Relative mRNA expression was calculated using the following equation: [A]_0_/[B]_0_ = E^ΔCt^, where [A]_0_ is the initial mRNA concentration of the target gene, [B]_0_ is the initial mRNA concentration of GAPDH, E is the reaction efficiency (1.98), and ΔCt is the difference between the Ct of GAPDH and the target gene. Calculations were performed using Microsoft Excel 2016. To facilitate data presentation and visualization, the resulting relative expression values were multiplied by 10^5^. Primers pairs were designed using PrimerBLAST (Primer3 version 2.5.0, NCBI, Bethesda, MD, USA). The primer sequences used in this study are listed in Table 2.

### 4.8. Statistical Analysis

Statistical analyses were performed using GraphPad Prism version 9.1 (GraphPad Software, Boston, MA, USA) and R. Data normality was assessed using the Shapiro–Wilk test. Since the data were not normally distributed, nonparametric statistical methods were applied. Comparisons among multiple groups were performed using the Scheirer–Ray–Hare test followed by Dunn’s post hoc test with Benjamini–Hochberg correction for multiple comparisons. For primary endpoints (behavioral changes, altered neuron morphology, Iba1+ microglia number) size effects were additionally evaluated. Data were presented as the median (Me) with the interquartile range (25%; 75%). Differences were considered statistically significant at *p* < 0.05.

## 5. Limitations

Several limitations of the present study should be acknowledged. The study was performed in 12-month-old male and female rats from control and prenatally LPS-exposed groups. Although both sexes were included, the analysis was restricted to a single age point. Therefore, the present results do not allow us to determine whether the observed morphological and molecular alterations emerge earlier in life, persist throughout aging, or progress with age. Longitudinal studies including multiple developmental and aging time points will be required to address the temporal dynamics of these changes.

The relatively small sample size should also be considered when interpreting the present findings. Each experimental group comprised seven animals. This sample size was determined in the context of a prolonged experimental protocol requiring the generation and maintenance of a sufficiently large number of litters. Particular attention was given to minimizing potential litter effects and pseudoreplication by obtaining offspring from multiple independent litters rather than increasing the number of offspring derived from the same litter. The long duration of the experiment, from prenatal exposure to 12 months of age, substantially increased the logistical requirements for maintaining independent litters and experimental animals. Consequently, the sample size may have provided limited statistical power for detecting relatively small effects in some morphological or molecular parameters. Therefore, the absence of statistically significant differences for individual outcomes should not necessarily be interpreted as evidence of the absence of a biological effect. Future studies with larger sample sizes, while retaining the litter as an important experimental unit and accounting for the hierarchical structure of the data, will be necessary to confirm and more precisely estimate the magnitude of the observed effects.

To minimize the potential influence of sex-dependent hormonal fluctuations, biological material from females was collected during diestrus, when circulating sex hormone levels are relatively low and more comparable to those in males. However, this approach does not completely eliminate the potential effects of gonadal hormones on gene expression. Future studies should therefore consider a more comprehensive assessment of sex-dependent hormonal and molecular factors.

The morphological analysis was intentionally limited to neuronal and microglial characteristics. This approach was selected as an initial exploratory assessment to determine whether prenatal LPS exposure was associated with persistent structural alterations in the brain at 12 months of age. The detection of group differences provides a basis for further investigation, but morphological changes alone are insufficient to establish the presence or severity of specific neurodegenerative processes or a particular state of microglial activation. In subsequent studies, we will employ an expanded panel of cellular and molecular markers to characterize neurodegenerative changes and distinguish pro-inflammatory microglial activation from other microglial phenotypes.

The molecular analysis was restricted to the expression of selected genes associated with the pro-inflammatory background, amyloid and tau metabolism, neuroprotection, and neuronal differentiation. These targets were selected as candidate pathways potentially affected by prenatal LPS exposure and were intended to provide an initial indication of the molecular processes that may underlie the observed morphological alterations. However, changes in transcript abundance do not necessarily reflect corresponding changes at the protein level or functional activity. Thus, the present gene-expression findings should be considered hypothesis-generating rather than definitive evidence of altered pathway activity. Based on the candidate pathways identified in the current study, future work will include the quantitative assessment of corresponding proteins and, where appropriate, additional functional and biochemical analyses to validate the proposed molecular mechanisms.

Overall, these limitations reflect the exploratory nature of the present study. The findings provide an initial morphological and molecular characterization of long-term consequences of prenatal LPS exposure in middle-aged rats and identify candidate cellular and molecular pathways for subsequent targeted investigation.

## 6. Conclusions

The present study demonstrates that a maternal immune activation during late pregnancy might be associated with persistent, sex-dependent alterations in hippocampal structure, microglial phenotype, gene expression, and behavior patterns that remain detectable at 12 months of age. Importantly, the long-term consequences of prenatal LPS exposure were not uniform between sexes. Female offspring displayed a phenotype characterized by greater neuronal vulnerability, a shift toward a more reactive microglial morphology, reduced *Bdnf* expression, and increased expression of *Bace1* and *Usp11*, whereas males showed predominantly ramified microglia together with increased *Pax6* expression, potentially suggesting the engagement of compensatory neuroprotective and neuroplastic mechanisms. At the same time, the persistent upregulation of *Il18*, *Arg1*, *Mmp9*, *Ntrk2*, and *Gpx4* in both sexes indicates that prenatal immune activation might establish a long-lasting alteration of the hippocampal molecular environment rather than a transient inflammatory response.

The consequences of prenatal inflammatory exposure might lead to not only developmental or early-life abnormalities, but also sex-specific increased vulnerability that persists into middle age. This supports the use of prenatal immune activation models as a framework for studying how early inflammatory events could modify the trajectory of later-life brain aging and susceptibility to neurodegenerative processes. In particular, the combination of microglial morphological alterations with changes in pathways related to amyloid metabolism, neurotrophic signaling, oxidative stress, and neuronal differentiation suggests candidate biological processes that can be targeted in subsequent mechanistic studies.

From a translational perspective, the results suggest that sex should be considered when searching for early molecular signatures of increased vulnerability to neurodegeneration following prenatal inflammatory exposure. The identified changes in *Bace1*, *Usp11*, *Bdnf*, *Ntrk2, Gpx4*, and *Pax6* provide a rational basis for selecting candidate markers for further validation rather than representing definitive biomarkers at this stage. The next step will therefore be to determine whether the observed transcriptional changes are accompanied by corresponding alterations at the protein and functional levels and whether they can predict subsequent progression of neuronal and microglial dysfunction.

Thus, the present work established a sex-dependent experimental framework for identifying mechanisms that may determine whether the aging brain follows a predominantly vulnerable or compensatory trajectory after prenatal inflammatory exposure. Such an approach might greatly facilitate the identification of individuals at increased risk before overt neurodegenerative pathology develops and provide a rationale for developing sex-informed preventive and therapeutic strategies aimed at modulating pro-inflammatory background, preserving microglial homeostasis, and maintaining neuronal resilience.

## Figures and Tables

**Table 1:** Sex-dependent behavioral, morphofunctional, and molecular changes in prenatally LPS-treated 12-month-old offspring.

m+lps	f+lps
**Performance in MWM**	
Preserved memory and spatial orientation functions and exploratory behavior	Suppressed exploratory behavior
**Performance in EPM**	
Decreased time spent in opened arms Increased anxiety index	Decreased distance moved
**Hyperchromic pyknotic neurons in the** **hippocampus**	
Increased in CA1 subregion	Increased sum number in all hippocampal subregions
**Iba1+ microglia in the hippocampus**	
Increased total number of cells Increased number of ramified cells	Decreased total number of cells * Decreased number of ramified cells *
**GFAP+ astrocytes in the hippocampus**	
No differences	No differences
**Genes expression in the hippocampus**	
**Associated with inflammation**	
Upregulation of *Il18*, *Arg1*, and *Mmp9*	Upregulation of *Il18*, *Arg1*, and *Mmp9*
**Associated** **with neuronal dysfunction**	
No changes	Upregulation of *Bace1* and *Usp11*
**Associated with neuronal protection**	
Upregulation of *Ntrk2* and *Gpx4*	Upregulation of *Ntrk2* and *Gpx4* Downregulation of *Bdnf*
**Associated** **with neurogenesis**	
Upregulation of *Pax6*	No changes

By default, differences are counted between control and LPS-treated groups of the same sex. *—Differences between LPS-treated male and female rats.

**Table 2:** Primer sequences used in qPCR-RT analysis.

Encoded Gene	Forward Primer Sequence (5′→3′)	Reverse Primer Sequence (5′→3′)
*App*	TGGATGATCTCCAACCGTG	CGTCGACAGGCTCAACTTC
*Bace1*	GGGCAGTAGTAATTTTGCAGT	TTCGGAGGTCTCGGTATGT
*Usp11*	AGTGTACCCAATAGAGCTGC	TTGCAAGACTAGGTCGACAG
*Bdnf*	CGAAGAGCTGCTGGATGAG	ATGGGATTACACTTGGTCTCG
*Ntrk2*	CACACACAGGGCTCCTTA	AGTGGTGGTCTGAGGTTGG
*Gpx4*	AATTCGCAGCCAAGGACATCG	ATTCGTAAACCACACTCGGCGTA
*Mmp9*	ATGGTTTCTGCCCCAGTGAG	CACCAGCGATAACCATCCGA
*Il18*	GACAAAAGAAACCCGCCTG	ACATCCTTCCATCCTTCACAG
*Pax6*	TGCAGGTGTCCAACGGATGT	CCAAGCAAAGATGGACGGGC
*Neurod1*	CCCGAGGCTCCAGGGTTATG	ACTCGTCTGTCCAGCTTGGG
*Arg1*	CGAGTCAGAGGTTTCACAGAG	CAATTCAGAGTCCCCTTCTGTC
*Gapdh*	GCGAGATCCCGCTAACATCA	CCCTTCCACGATGCCAAAGT

## Data Availability

The original contributions presented in this study are included in the article. Further inquiries can be directed to the corresponding author(s).
